# Hypoglycaemic treatment adherence and the association with psychological, self‐management and glycaemic characteristics in adults with type 1 diabetes

**DOI:** 10.1002/nop2.267

**Published:** 2019-03-20

**Authors:** Pamela Martyn‐Nemeth, Reid Birlingmair, Esema Idemudia, Chang Park

**Affiliations:** ^1^ Department of Biobehavioral Health Science, College of Nursing University of Illinois at Chicago Chicago Illinois; ^2^ Department of Health Systems Science, College of Nursing University of Illinois at Chicago Chicago Illinois

**Keywords:** depressive mood, glycaemic control, glycaemic variability, hypoglycaemia, stress, type 1 diabetes

## Abstract

**Aim:**

The purpose of this study was to examine adherence to hypoglycaemia treatment guidelines in adults with type 1 diabetes (T1DM). The American Diabetes Association recommends consumption of 15–20 g of glucose to treat hypoglycaemia. Overtreatment may result in poor glycaemic control and greater glycaemic variability. It is not fully understood how well T1DM adults comply with hypoglycaemia treatment recommendations.

**Design:**

A secondary analysis using a descriptive comparative design.

**Methods:**

Using real‐time measures over six consecutive days, we examined (a) adherence to hypoglycaemia treatment guidelines and (b) comparisons of demographic self‐management behaviour, psychological characteristics and glycaemia between adherent and non‐adherent groups.

**Results:**

Findings revealed those who overtreated consumed more daily grain servings and reported higher stress and depressed mood compared with those who followed treatment recommendations. Findings suggest that hypoglycaemia treatment practices and psychological factors influencing self‐management should be assessed.

## 1 INTRODUCTION

Achieving recommended glycaemic targets is associated with a reduction in micro‐ and macrovascular complications in type 1 diabetes (T1DM; Nathan et al., 2005). Hypoglycaemia, defined as a blood glucose level ≤70 mg/dl (3.9 mM; Seaquist et al., 2013), is a major limiting factor in achieving recommended glycaemic targets. For individuals with T1DM, hypoglycaemia occurs frequently and has been reported to occur approximately once per day (Martyn‐Nemeth et al., 2017). The American Diabetes Association (2018) recommends consumption of 15–20 g of carbohydrate (CHO) in conscious individuals to treat a blood glucose level of ≤70 mg/dl. Overtreatment may result in poor glycaemic control and greater glycaemic variability (the intraday fluctuation in blood glucose). Current reports indicate that only 13%–29% of adults meet the glycaemic target of HbA1c <7% (Miller et al., 2015). If overtreatment of hypoglycaemia contributes to the inability to meet glycaemic goals, it would be important to have a better understanding of contributing factors.

## 2 BACKGROUND

Few studies have examined adherence to hypoglycaemia treatment recommendations. Those conducted reported overtreatment in 38%–73% of mild to moderate hypoglycaemic episodes (Banck‐Petersen et al., 2007; Savard et al., 2016). Gender differences were reported, with women more compliant with treatment recommendations than men (Banck‐Petersen et al., 2007). Those who overtreated were significantly younger (Savard et al., 2016).

Psychological factors such as stress and fear of hypoglycaemia (FOH) also play a contributory role in overtreatment. Savard et al. (2016) reported greater FOH in those who overtreated. Descriptions of how FOH negatively influenced self‐management behaviour were reported by Lawton et al. (2013), who followed participants for 1 year after completion of a diabetes education programme. Immediately after the programme, participants were motivated to follow treatment guidelines, but over time, the memories of previous frightening hypoglycaemic events made it difficult to adhere to treatment recommendations. Many participants reverted to their previous patterns of overtreatment because of the anxiety felt when remembering past negative hypoglycaemic experiences (Lawton et al., 2013). Depression is linked with poorer self‐management behaviours (Ahola & Groop, 2013) and thus might influence hypoglycaemia treatment patterns. To our knowledge, the association of mood with hypoglycaemia treatment has not been investigated.

Consistent overtreatment theoretically may worsen glycaemic control. The association of overtreatment with glycaemic control was examined in one study, with no statistically significant differences with overtreatment observed (Savard et al., 2016). Further investigation is needed to compare hypoglycaemia treatment behaviour with glucose measures. Overtreatment is problematic because it may result in rebound hyperglycaemia and greater glycaemic variability. Glycaemic variability has been associated with diabetes complications (Soupal et al., 2014), endothelial dysfunction (Ceriello et al., 2012) and cardiovascular events (Yoon et al., 2016).

In summary, overtreatment of hypoglycaemia occurs frequently among adults with T1DM. FOH plays a role in overtreatment despite adults with T1DM having adequate knowledge of how to treat hypoglycaemia. The associations of self‐management behaviours, psychological factors, glycaemic control and glycaemic variability have not been adequately explored. The purpose of this secondary analysis was to (a) examine adherence to hypoglycaemia treatment guidelines and (b) compare demographic data, self‐management behaviour (diet, eating behaviour, insulin dosage), psychological factors (stress, depressive mood, FOH) and glycaemia measures (glycaemic control and glycaemic variability) between adherent and non‐adherent groups. The research questions were as follows:

1. What is the adherence to hypoglycaemia treatment guidelines among those with T1DM?
2. How do demographic, self‐management behaviour, psychological factors and glycaemic parameters differ between those who adhere versus those who do not adhere to hypoglycaemia treatment guidelines?

## 3 THE STUDY

### 3.1 Design

A descriptive comparative design was used. This was a secondary analysis of data from a parent study (*N* = 39) that sought to determine temporal associations of FOH with glycaemic variability in young adults with T1DM.

### 3.2 Method

In the original study, questionnaires (demographic, self‐management and psychological characteristics); measured height and weight; and haemoglobin A1C were collected at an initial visit for individuals aged 18–39, who had T1DM for at least 1 year and used an insulin pump. Insulin pump downloads, daily diary and continuous glucose monitoring data were collected over six consecutive days. Data were collected 2014–2016.

In this secondary analysis, we (a) examined adherence to hypoglycaemia treatment guidelines and (b) compared demographic data, self‐management behaviour (diet, eating behaviour, insulin dosage), psychological factors (stress, depressive mood, FOH) and glycaemia measures (glycaemic control and glycaemic variability) between adherent and non‐adherent groups.

### 3.3 Variables and measures

#### 3.3.1 Participant characteristics

Demographic, diabetes and treatment regimen characteristics were obtained by self‐report. Measured height and weight were used to calculate body mass index.

#### 3.3.2 Hypoglycaemia

Episodes of hypoglycaemia and subsequent treatments were recorded in a daily diary over six consecutive days. Participants recorded the date, time, blood glucose level, possible cause of the episode and subsequent treatment. Diaries were analysed for episodes of hypoglycaemia and associated treatment and verified with insulin pump downloads. An episode of hypoglycaemia was defined as a blood glucose level ≤70 mg/dl (3.9 mM) with or without symptoms, or <90 mg/dl (5 mM) if symptoms were present (Savard et al., 2016). Participants were categorized as adherent (those who treated with 15–20 g per ADA guidelines [2018]) or non‐adherent (those who treated with <15 g [undertreatment] or >20 g [overtreatment]).

#### 3.3.3 Self‐management behaviour

##### Dietary patterns and eating behaviour

Usual dietary patterns over the past year were measured with the Block Food Frequency Questionnaire^®^ (FFQ; Block, Woods, Potosky, & Clifford, 1990), a 110‐item survey of the frequency and portion size of usual dietary intakes of foods, nutrients and food groups. It was developed from the NHANES dietary recall (1999–2002) and the USDA nutrient databases and has been validated using concurrent dietary recall (Block et al., 1990).

Eating behaviour was obtained with the 51‐item Three‐Factor Eating Questionnaire (TFEQ), which measures three components of eating behaviour: dietary restraint (conscious restriction of food intake), disinhibition (emotional stress‐induced eating behaviour) and hunger (dietary intake in response to hunger). The scale has been psychometrically validated (Cronbach's alpha: 0.79–0.93; concurrent validity attained) (Stunkard & Messick, 1985). Higher scores on each subscale indicate greater endorsement of each domain. Scores >7 on the restraint and disinhibition subscales indicate high levels of that characteristic (Lesdema et al., 2012).

##### Daily CHO intake and insulin dosage

Participants were instructed to input all CHO intake into their insulin pumps each time CHOs were ingested. Total daily CHO intake and insulin doses were downloaded from each participant's pump on Day 6.

#### 3.3.4 Psychological measures

##### Stress

Stress was measured with the Perceived Stress Scale. This 10‐item, 5‐point Likert‐style scale measures generalized life stress perceptions over the past month. The scale has a unidimensional factor structure and has strong reliability and validity (Cronbach's alpha: 0.83–0.86 and construct, concurrent and predictive validity are established; Cohen, Kamarck, & Mermelstein, 1983). Higher scores indicate greater stress.

##### Depressive mood

Depressive mood was measured with the 20‐item, 4‐point Likert‐style Center for Epidemiologic Studies Depression Scale (CES‐D). This scale rates symptoms of depressive mood experienced over the past week. Higher scores indicate greater depressive moods, and scores ≥16 indicate risk of depressive mood (Vilagut, Forero, Barbaglia, & Alonso, 2016).

##### Fear of hypoglycaemia

Fear of hypoglycaemia was measured with the 18‐item, 5‐point Likert‐style Worry Scale of the Hypoglycemia Fear Survey II. This scale measures the frequency of worries about hypoglycaemia in persons with diabetes. The scale has been psychometrically validated (Cronbach's alpha: 0.95; construct and convergent validity demonstrated; Gonder‐Frederick, Cox, & Vajda, 2011; Gonder‐Frederick, Schmidt et al., 2011). Higher scores indicate greater FOH frequency. The frequency of worries is totalled for an overall score. Worry item scores of 3 or 4, indicating that worry occurs *often* or *very often,* were used to determine the presence of FOH, as previously described (Hajos, Polonsky, Pouwer, Gonder‐Frederick, & Snoek, 2014).

#### 3.3.5 Glycaemic measures

##### Glycaemic control

Glycaemic control was measured using A1C, which provides the mean blood glucose level over the previous 2–3 months. This was done by obtaining a finger stick drop of blood using A1C Now^®^ (Polymer Technology Systems, Inc., Indianapolis, IN).

##### Glycaemic variability

Glycaemic variability was derived from interstitial glucose recordings measured continuously over 6 days using a continuous glucose monitor (CGM; iPro2^®^; Medtronic, Northridge, CA). The CGM was blinded so that participants could not view their glucose levels. Interstitial glucose levels were recorded at 5‐minute intervals, resulting in 288 readings per day. The CGM recordings were downloaded using Medtronic software and examined for trends. Glycaemic variability was calculated as the 24‐hr glucose standard deviation, as previously described (Rodbard, 2009).

### 3.4 Data collection

At the initial visit, participants completed questionnaires for demographic and diabetes characteristics, usual dietary patterns, usual hypoglycaemia treatment methods and psychological variables. A1C was measured, and the CGM was applied. Participants wore a CGM in their free‐living environment over six consecutive days and were instructed to keep a daily diary of hypoglycaemic events over the same period. The CGM site was changed at a study visit on the third day, per manufacturer guidelines. On the sixth day, participants returned for a final visit to have the CGM removed, diaries collected, insulin pumps downloaded and compensation provided.

### 3.5 Analysis

For this study, the data were screened for missing values and those cases were removed. Of the remaining 31 cases with no missing data, demographic, diabetes, self‐management, psychological and glycaemic measures were examined using descriptive statistics (SPSS 24) to characterize the sample.

One participant was categorized into the undertreatment group. Due to this small number, group comparisons were conducted using adherent and overtreatment groups only. A Mann–Whitney *U* test (for continuous variables) or chi‐square test (for categorical variables) was used to examine the demographic, diabetes, self‐management, psychological and glycaemic characteristics between these two groups. Due to the exploratory nature of this study, adjustments were not made for multiple comparisons.

### 3.6 Ethics

Institutional review board approval for the protection of human subjects was obtained from the University of Illinois at Chicago. Informed consent was obtained from each participant prior to data collection.

## 4 RESULTS

The final sample consisted of 31 adults, 18–39 years of age, diagnosed with T1DM for 1–35 years (mean = 14.3 *SD* 8.5). Most were female (71%), White (87%), single (61%), working full time or part time (62%) and had earned a minimum of a college degree (84%; Table 1). All had previously attended a diabetes self‐management programme.

**Table 1:** Participant characteristics

Demographics	Range	Mean ± *SD*	*N* (%)
Age (years)	18–39	26.6 ± 5.0	
Sex			
Female			22 (71)
Male			9 (29)
Race			
Black			2 (7)
White			27 (87)
Mixed			2 (7)
Ethnicity			
Not Latino			29 (94)
Latino			2 (6)
Marital status			
Single			19 (61)
Married			6 (19)
Living with other			5 (16)
Other			1 (3)
Education			
Finished high school			1 ( 3)
Some college			4 (13)
College			19 (61)
Master's or more			7 (23)
Work			
Full time			17 (55)
Part time			2 (7)
Full‐time student			7 (23)
School and work			5 (16)
Health			
Diabetes duration (years)		14.3 ± 8.5	
Hypoglycaemic unawareness		2 ± 6	
Body mass index		27 ± 4.2	
A1C (%)		7.4 ± 1.0	

### 4.1 Hypoglycaemia

During the 6‐day period, 158 hypoglycaemic episodes were recorded by the 31 participants. All participants experienced hypoglycaemia. The mean number of episodes over the 6 days was 5.1 (*SD*: 3.3; range: 1–12). At the daily level, participants experienced 1–4 hypoglycaemic episodes per day (mean* = *0.80 episodes/day).

### 4.2 Self‐management behaviour

One person (3%) undertreated their hypoglycaemic episodes, 16 (52%) were adherent and 14 (45%) overtreated. The most frequent method for treating hypoglycaemia was ingestion of candy, sugar or glucose tablets. Those who were treated in guidelines primarily used small pre‐packaged candy or glucose tablets with easily identified CHO gram levels to facilitate consistent treatment. In terms of dietary patterns, the average usual dietary intake for the entire sample was comprised of 40% fat, 16% protein and 42% CHO. Mean scores on the TFEQ revealed high levels of dietary restraint (mean = 9.1 *SD* 5.1) and lower levels of disinhibition (mean = 6.4 *SD* 3.6) and susceptibility to hunger (mean = 5.8 *SD* 3.5).

### 4.3 Psychological measures

Measured stress, depressive mood and FOH levels were normally distributed. Twelve of the 31 participants (39%) had stress levels above the normed mean for the general population (16.9; Cohen & Janicki‐Deverts, 2012). Five (16%) had depressive moods indicative of risk of depression, while 24 (77%) experienced high levels of FOH.

### 4.4 Glycaemic measures

The mean A1C was 7.4% (*SD* 1%); 19 participants (61%) had A1C ≥ 7%. The mean time spent in hyperglycaemia (>180 mg/dl) was 7.4 hr per day. Glucose variability ranged from 27–99 mg/dl (mean = 57 *SD* 17).

### 4.5 Comparisons between groups

Comparisons between adherent and overtreatment groups revealed that those who overtreated had significantly higher mean stress and depressive mood levels (mean = 11.9 [*SD* 5.6] vs 17.6 [*SD* 4.1], *p* = 0.004; and 6.8 [*SD* 5.1] vs 11.7 [*SD* 5.8], *p* = 0.016, respectively). They also ate significantly more servings from the grain group (mean = 3.1 *SD* 1.7 vs 5.6 *SD* 3.1, *p* = 0.022). Those who were adherent exhibited more dietary restraint, whereas those who overtreated exhibited more disinhibited eating behaviour, although these differences were not statistically significant (Table 2). Total daily insulin dose, glycaemic control and glycaemic variability were not significantly different between the groups (Table 2).

**Table 2:** Differences in participant characteristics between adherent and overtreatment groups (mean ± *SD*)

Characteristic	Adherent (*N* = 16)	Overtreatment (*N* = 14)	*p* value	95% Confidence interval	Effect size (Cohen's *d*)	Post hoc power calculation
Age (years)	26.13 ± 4.6	26.7 ± 5.4	0.785	0.791−0.806	0.114	0.139
Diabetes duration (years)	14.4 ± 8.3	13.2 ± 8.2	0.466	0.470−0.490	0.145	
Body mass index	26.6 ± 3.7	26.6 ± 3.5	0.803	0.805−0.821	0	
Hypoglycaemic episodes	5.4 ± 3.2	4.9 ± 3.6	0.502	0.510−0.530	0.147	0.180
Total daily insulin (u/kg)	0.55 ± 0.18	0.72 ± 0.32	0.160	0.162−0.176	0.655	0.946
Carbohydrate (g/day; diary derived)	140 ± 44	178 ± 69	0.197	0.200−0.216	0.657	0.947
Carbohydrate (g/day; FFQ‐derived)	156 ± 44	201 ± 84	0.280	0.281−0.299	0.671	0.954
Protein (g/day)	63 ± 19	73 ± 28	0.280	0.281−0.299	0.418	0.667
Fat (g/day)	66 ± 22	83 ± 23	0.070	0.064−0.074	0.755	0.982
Fruit servings	1.8 ± 0.87	1.1 ± 0.9	**0.008**	**0.006−0.009**	**0.791**	**0.989**
Vegetable servings	3.3 ± 1.4	2.4 ± 1.5	0.088	0.085−0.097	0.620	0.924
Grain servings	3.1 ± 1.7	5.6 ± 3.1	**0.022**	**0.017−0.023**	**0.999**	**0.75**
Meat servings	2.2 ± 1.0	2.5 ± 1.1	0.318	0.323−0.341	0.285	0.409
Dairy servings	1.1 ± 0.7	1.3 ± 1.0	0.950	0.956−0.964	0.232	0.311
Dietary restraint	10.4 ± 5.3	8.0 ± 4.5	0.242	0.235−0.252	0.488	0.782
Dietary disinhibition	5.0 ± 3.1	7.5 ± 3.2	0.051	0.046−0.054	0.794	0.793
Hunger	4.7 ± 3.2	6.6 ± 3.1	0.103	0.098−0.110	0.603	0.911
Stress	11.9 ± 5.6	17.6 ± 4.1	**0.004**	**0.002−0.005**	**1.16**	0.86
Depressive mood	6.8 ± 5.1	11.7 ± 5.8	**0.016**	**0.011−0.015**	**0.897**	0.657
Fear of hypoglycaemia	24.4 ± 12.5	29.4 ± 11.8	0.228	0.229−0.246	0.411	0.654
A1C (%)	7.3 ± 1.0	7.6 ± 1.1	0.580	0.563−0.582	0.285	0.409
Glycaemic variability (Gluc *SD*)	55.4 ± 15.3	56.9 ± 17.7	0.454	0.465−0.484	0.091	0.116

Mann–Whitney *U* test with Monte Carlo simulation for confidence intervals. Bold values are statistically significant (*p* < 0.05).

## 5 DISCUSSION

Findings indicated that overtreatment of hypoglycaemia occurred frequently among adults with T1DM. Nearly half (45%) overtreated beyond ADA recommendations and those who did consumed more grain servings than those who were adherent. Those who overtreated also had higher levels of stress and depressive moods than those who were adherent. A post hoc analysis revealed that the effect sizes between the two groups were 1.16 and 0.897, respectively, to detect a difference in each: (a) stress; (b) depressive mood; and (c) grain intake.

The prevalence of overtreatment is consistent with previous studies that observed overtreatment in 39%–78% of participants (Banck‐Petersen et al., 2007; Larsen et al., 2006; Savard et al., 2016). We did not observe age or gender differences as did Banck‐Petersen et al. (2007) and Savard et al. (2016), respectively; however, our sample was mostly young and female.

As expected, those who were adherent participated in more dietary restraint and those who overtreated showed more disinhibited eating behaviours. Disinhibition is a stress‐induced eating style where heightened stress and emotion contribute to overconsumption of food. It is closely associated with weight gain in the general population (Lesdema et al., 2012), overeating in women with type 2 diabetes (van de Laar et al., 2006) and emotional distress in women with T1DM (Martyn‐Nemeth, Quinn, Hacker, Park, & Kujath, 2014). The stressors associated with food intake among those with T1DM are considerably different from in the general population, particularly as they relate to treatment of hypoglycaemia; thus, they require further investigation. Greater total CHO intake was seen in the overtreatment group both on the FFQ and daily diary of CHO intake. If persistent, this behaviour could lead to greater weight gain over time.

What our study adds to the existing body of literature is the role of stress in overtreatment practices. FOH is a major stressor among persons with T1DM (Vallis, Jones, & Pouwer, 2014). In our sample, 77% experienced elevated FOH and 39% experienced generalized stress that was above the normed mean for the general population. The high level of FOH across groups may explain why we did not see a statistically significant difference in the overtreatment group; FOH likely affected both groups.

Stress has been linked with diabetes self‐management practices. Boden and Gala (2018) examined stress among 10,821 adults from the T1D Exchange and reported a high degree of both general and diabetes‐related stress, which contributed negatively to many self‐management practices, including responding to hypoglycaemia. Stress can impair decision‐making (Reach, 2013); conversely, poor decision‐making can result in heightened stress. Thus, a bidirectional relationship is plausible. While this study is cross‐sectional and we cannot address causation, coping with stress associated with managing hypoglycaemia is a potentially modifiable risk factor for improving self‐management behaviour and quality of life.

Depressive mood was also significantly greater among those who overtreated. These findings support the well‐established association of depression with poor self‐management (Schmitt et al., 2017). However, it may be important to investigate further the association of depression with hypoglycaemia management. In a prospective longitudinal study, Katon et al. (2013) reported that baseline depression predicted subsequent hypoglycaemia. The mechanisms for this have not been established.

No differences were observed between the two treatment groups in glycaemic control or glycaemic variability. One possible explanation is that individuals followed their glucose levels closely and adjusted for overtreatment‐related hyperglycaemia. All participants in this study used insulin pump therapy; thus, reducing the basal rate infusion is another approach to meeting glycaemic targets. It also raises the question of whether some people require more glucose to treat a hypoglycaemic episode. It is possible that participants in this study were aware of the amount of CHO needed to treat their hypoglycaemic episodes in the context of their activity, lifestyle and metabolic needs.

### 5.1 Limitations

Because this was a secondary analysis, the original study was not powered to address the comparisons made. However, a post hoc analysis revealed that the effect sizes between the two groups were 1.16 and 0.897, respectively, to detect a difference in each: (a) stress; (b) depressive mood; and (c) grain intake. Secondly, our sample, comprised of younger, well‐educated adults who used insulin pump therapy, may not reflect the overall T1DM population. In addition, we relied on participants to estimate correctly the CHO ingested and to enter it accurately into their insulin pumps and diaries. Lastly, within‐day variations in treatment were not examined to determine whether overtreatment tended to occur at a specific time of day.

## 6 CONCLUSION

In summary, adults in this study experienced a high frequency of hypoglycaemia and nearly half of the participants overtreated the hypoglycaemia. Findings suggest that treatment practices and psychological factors influencing hypoglycaemia self‐management should be addressed and investigated further.

It is important to assess the frequency and severity of hypoglycaemia episodes among adults with T1DM. Asking patients to maintain a diary to track the frequency, cause and treatment of hypoglycaemia may facilitate strategies to improve hypoglycaemia treatment practices when indicated and to support and reinforce behaviours when appropriate. Because stress and negative mood were linked with overtreatment, it would be important to evaluate stress levels and coping strategies used to address diabetes‐related concerns, and general life stress. The use of a stress‐induced eating style is also important to evaluate because it has been linked with weight gain and poor health outcomes. Consideration should be given to the possible links between the frequency of hypoglycaemia and meeting glycaemic targets. Our findings suggest that attempts to meet glycaemic targets may increase hypoglycaemia events.

## CONFLICT OF INTEREST

The authors declare no conflicts of interest.
